# A Total Randomized SLP-Preserving Technique with Improved Privacy and Lifetime in WSNs for IoT and the Impact of Radio Range on SLP

**DOI:** 10.3390/s23249623

**Published:** 2023-12-05

**Authors:** Florence Mukamanzi, Raja Manjula, Raja Datta, Tejodbhav Koduru, Damien Hanyurwimfura, Mukanyiligira Didacienne

**Affiliations:** 1 African Center of Excellence in the Internet of Things, University of Rwanda, CST, Kigali P.O. Box 3900, Rwanda; hadamfr@gmail.com (D.H.); dmukanyligira@ncst.gov.rw (M.D.); 2 Department of Computer Science and Engineering, SRM University AP, Amaravathi 522502, India; rajamanjula12@gmail.com (R.M.); tejkodur@gmail.com (T.K.); 3 Department of Electronics and Electrical Communication Engineering, Indian Institute of Technology Kharagpur, Kharagpur 721302, India; rajadatta@ece.iitkgp.ac.in

**Keywords:** radio range, network lifetime, SLP, safety period, uniform privacy, WSN

## Abstract

Enhanced source location privacy and prolonged network lifetime are imperative for WSNs—the skin of IoT. To address these issues, a novel technique named source location privacy with enhanced privacy and network lifetime (SLP-E) is proposed. It employs a reverse random walk followed by a walk on annular rings, to create divergent routing paths in the network, and finally, min-hop routing together with the walk on dynamic rings to send the packets to the base station (BS). The existing random walk-based SLP approaches have either focused on enhancing only privacy at the cost of network lifetime (NLT) or have aimed at improving the amount of privacy without degrading the network lifetime performance. Unlike these schemes, the objectives of the proposed work are to simultaneously improve the safety period and network lifetime along with achieving uniform privacy. This combination of improvements has not been considered so far in a single SLP random walk-based scheme. Additionally, this study investigates for the first time the impact of the sensors’ radio range on both privacy strength and network lifetime metrics in the context of SLP within WSNs. The performance measurements conducted using the proposed analytical models and the simulation results indicate an improvement in the safety period and network lifespan. The safety period in SLP-E increased by 26.5%, 97%, 123%, and 15.7% when compared with SLP-R, SRR, PRLPRW, and PSSLP techniques, respectively. Similarly, the network lifetime of SLP-E increased by 17.36%, 0.2%, 83.41%, and 13.42% when compared with SLP-R, SRR, PRLPRW, and PSSLP techniques, respectively. No matter where a source node is located within a network, the SLP-E provides uniform and improved privacy and network lifetime. Further, the simulation results demonstrate that the sensors’ radio range has an impact on the safety period, capture ratio, and the network lifetime.

## 1. Introduction

Internet of Things (IoT) research has been considered as a paramount domain while enhancing current technologies such as wireless sensor networks (WSNs) [1]. To assist individuals in finding information in various fields, IoT deployment intends to collect data via a wireless connection. IoT is easily deployed in locations with widespread internet access. However, in wilderness areas such as wildlife sanctuaries, military battlefields, polar regions of the earth, etc., internet connectivity is inadequate or unreliable. In such cases, WSNs play a key role in mitigating this issue. The sensor nodes are primarily used to monitor and track priceless assets, such as reporting in-the-moment military data from a battleground, tracking the whereabouts of threatened species in their habitats [2,3,4,5,6,7], etc. In particular, the sensors form a mesh network and send the collected data wirelessly to single or multiple central controllers known as base stations (BSs).

It is observed that establishing a direct communication link between IoT nodes and the sink node leads to a noteworthy decrease in the remaining energy levels of the individual nodes [8]. Therefore, within the context of IoT applications, ensuring an adequate battery charge within the IoT nodes holds significant importance. Various research initiatives are dedicated to addressing challenges associated with energy consumption and the prolonged lifetime of networks. Singh et al. in [9] endeavored to address concerns regarding the optimal placement of RF-energy transmitters by introducing a network-aware scheme for positioning these transmitters. This approach becomes pivotal, as radio frequency (RF) stands out as one of the primary ambient energy sources viable for energy harvesting. Zheng et al. conducted an in-depth study on the RF-powered AB-assisted hybrid underlay cognitive radio network (ABHU-CRN), with the aim of enhancing the long-term secondary throughput in [10]. This research endeavor was undertaken to tackle the critical challenges posed by energy and spectrum constraints within wireless sensor networks. Numerous approaches have been introduced by various researchers to enhance the operational lifespan of networks. These methods encompass optimal power schemes, as outlined in Reference [11], which facilitate a systematic utilization of the nodes’ remaining energy. Additionally, the clustering of IoT nodes, as discussed in Reference [12], has been employed. Furthermore, intelligent energy utilization approaches, such as innovative routing techniques and the application of wake-up radio protocols detailed in References [13,14], have also contributed to enhancing network lifetime. Since these WSNs are deployed in internet-devoid zones, the BS is supposed to be connected to an IoT gateway that is situated in common places where an internet facility is available. Data collected from the WSN can then be forwarded to the operator, via IoT for live tracking and monitoring purposes.

However, alongside network lifetime, there are other undesirable issues associated with these networks. The nature of wireless communication channels makes security and privacy crucial considerations for these networks. The attacker may employ *active attacks* such as packet dropping or *passive attacks* such as eavesdropping on the signals. Several solutions have been proposed in the literature by various researchers to mitigate such attacks [6,15,16,17,18,19,20,21,22]. Source location privacy (SLP) protection techniques are the name given to these solutions.

One such domain we explore is contextual privacy [7]—it deals with hiding contextual information such as packet rate, routing paths, identities of sensor nodes, etc. Particularly, this paper aims at protecting the source location information from the backtracking attacker. We consider wildlife applications where sensor nodes are deployed to keep track of endangered animals such as rhinos, pandas, tigers, etc. In this scenario, the hunter plays the role of the attacker who attacks the network from the base station (BS) and backtracks the signals to reach the source of information. The term “source node” refers to a sensor node that recognizes an asset (in this case, an animal) and communicates the asset information to the BS utilizing mesh-type routing. Similar to this, the replacement of a dead node in the network is extremely difficult or impossible since the sensor nodes are deployed in a hostile and harsh environment. Therefore, when developing a new routing protocol, the network lifetime (NLT) and privacy should be given careful consideration since prolonging network lifetime enables long-term asset monitoring.

The current SLP solutions in the literature can be categorized broadly into two distinct categories, namely, phantom routing (random walk)-based SLP and fake packets/fake source-based SLP techniques [23,24]. In the former case, random walk techniques are employed to randomize the routing paths, and in the later scenarios, dummy packets or fake sources are employed to introduce anonymity and unobservability features to the original traffic. These approaches aim to obfuscate the attacker.

The approach suggested by Long et al. in [22] improved the safety period by utilizing both phantom nodes and fake sources by using a tree-based diversionary routing approach with diversionary routes as its branches. The fake nodes are employed at the end of diversionary pathways to further confuse an attacker. Han et al. in [25] proposed a scheme that employs multiple sinks and switches the packet destination at random with each transmission to trick the attacker. A cloud-shaped fake hotspot was created to introduce fake packets into the network to confuse the adversary and increase privacy. The intermediate node was utilized to make the routing path more unpredictable. In [26], researchers suggested a technique that utilized phantom nodes and fake packets to enhance privacy without compromising NLT. This strategy takes neighbor nodes’ weight into account while choosing the next hop to relay the packet, in contrast to the previous ones. The work in [27] suggested a fake packet/fake sources-based SLP scheme which aimed at enhancing the safety period while minimizing packet transmission latency. It can be noted that these schemes may provide enhanced privacy; however, employing fake sources may affect the network’s lifetime since the fake sources keep sending fake packets to the network. The greater the frequency with which specific nodes within the network transmit and receive fake packets, the more their energy is decreased, consequently exerting a detrimental impact on the overall network’s lifetime. Our research work focuses on the use of random walk-based routing techniques and their variants to provide SLP in WSNs, as fake packets/fake source approaches are energy-expensive and are not advisable for resource-constrained sensor networks.

Various random walk SLP schemes have been proposed in the literature; authors in [28] proposed a novel random walk-based scheme that involves the segmentation of the network into distinct sectors. The selection of a particular sector is determined at random for every new packet originating from the source node. Subsequently, these packets proceed towards their designated sector, ultimately making their way to the base station (BS). The article [29] introduced a phantom nodes-based scheme. In this scheme, the source node selects a predefined set of network nodes to act as phantom nodes. The packets originating from the source node follow a path through these selected phantom nodes to reach the ring node and, subsequently, to be sent to the base station (BS). Another random walk-based solution was suggested by Li et al. in [30]. Their scheme enhances the privacy level of the source node by setting two candidate domains and the proxy node that will serve as the source node to confuse an adversary. In [20], authors proposed a bidirectional location-based SLP scheme to protect both source and sink node. More existing random walk-based SLP solutions, their contributions, and their weaknesses are detailed in Section 2.

Nevertheless, we noticed that the existing random walk-based solutions in SLP have either focused only on enhancing privacy, i.e., the safety period (see Section 6 for the definition) at the cost of reduced network lifetime, or have focused on enhancing privacy without hampering the network lifetime (NLT). However, enhancing both privacy and network lifetime together in a single solution does not exist in the literature. The objective of this work is to present a new random walk-based SLP routing protocol (SLP-E) that offers consistent privacy and improves both the safety period and network lifetime. No existing SLP solution based on random walks exhibits these combined improvements in the literature. This study also looks into how the radio range of sensor nodes affects network lifetime and privacy strength, which is not investigated in the existing SLP research. The existing works in the literature that tried to enhance both safety period and NLT in a single solution do not belong in the random walk-based category. The scheme suggested by the authors of [22] tried to maximize both the safety period and the network lifetime in a single solution. In addition to the introduction of phantom nodes in their scheme, fake sources are used to confuse a backtracking attacker to enhance privacy. In order to enhance NLT, the scheme tries to reduce energy consumption in the hotspot area (near the sink) by establishing diversionary routes that only use a lot of energy in non-hotspot areas. However, the introduction of fake sources can affect the NLT. Furthermore, it has been observed that their scheme does not take into account the energy level as a criterion when selecting a node from the forwarding group to relay the packet. Consequently, if nodes with lower energy continue to relay the packets, their batteries may eventually become depleted, resulting in a poor network lifetime (NLT). The work in [31] also proposed a scheme that improves both the privacy level and the network lifetime in a single solution, but the aim of this study is to mitigate the issues of black hole attacks and not to focus on SLP.

In addition, we have also observed that the safety period in existing phantom routing schemes (random walk-based-SLP) exhibits a distance-dependent behavior, i.e., the closer the source node is to the BS, the smaller the safety period is. Further, as the distance between the source node and the BS becomes larger, the safety period value also becomes larger. The attacker might use this behavior to infer the asset information such as the location information. The proposed scheme (SLP-E) aims to address this problem by offering a constant level of privacy protection regardless of where the source node is located within the network, achieving enhanced privacy and network lifetime. Also, this work aims at answering the long-awaited question, i.e., “*Is there any impact of sensors’ radio range on safety period and NLT*” in SLP solutions? For achieving a prolonged network lifetime of the WSNs, a study was made in [31] to see the impact of sensors’ radio range on the network lifetime. However, this work was not aimed at source location privacy (SLP). To the best of our knowledge, no research exists in the literature that aims at SLP that has studied the impact of the sensors’ radio range on privacy level (safety period) and NLT.

This article proposes a novel scheme that takes into account the backtracking attacker that initiates its backtracking process from the BS, as it is the final destination for all incoming packets, like in Ref. [22]. The proposed technique is referred to as source location protection with enhanced privacy and network lifetime (SLP-E). In SLP-E, the packets are transmitted to the base station in a controlled random walk manner to ensure a better safety period and network lifetime without injecting fake packets/fake sources. The routing path is random and diverse enough to confuse a backtracking attacker. To balance the distance they cover before reaching the BS, the packets take several routes. To establish fairness between the network lifespan (also known as network lifetime) and safety period, the hop threshold notion is presented. A random neighbor is chosen from the far-off neighbor list to relay a packet. This choice is based on the quantity of residual energy present in the nodes that are on the list of far neighbors. Like the walk in a game of a maze, the packets initially travel in a backward direction from the source for specific hops before taking clockwise or anti-clockwise paths across the annular rings of the network. In this stage of the routing process, the routing paths are lengthened to decrease the likelihood that packets will travel across the source node’s radio range (known as the visible area). SLP-E then uses controlled walks on dynamic annular rings that depend on the location of the source node in the network with respect to the BS along with min-hop routing to send the packet in the inward direction to reach the BS, in contrast to the existing solutions where packets follow only min-hop (shortest path) or forward random walks in the inward direction to reach the BS. The controlled walks on the dynamic annular rings in the inward direction help to increase the randomness and diversity of the routing path, which subsequently results in the usage of different sets of nodes for a packet on its way to the BS. Thus, the improvement in network lifetime and the safety period. The utilization of only min-hop routing in the inward direction seen in the existing solutions may result in the usage of the same set of nodes, which may weaken the privacy level and affect NLT. This technique (SLP-E) places a strong emphasis on enhancing the dynamism of the routing path. Its primary goal is to ensure that data packets traverse various routes, often referred to as dynamic routes, when traveling towards the base station (BS). This strategic diversification of routing paths serves the purpose of preventing packets from repeatedly traversing the same route (visiting the same nodes) to the BS, which can lead to rapidly depleting energy resources within those particular nodes, ultimately leading to a shortened network lifetime (NLT). Consequently, as the routing path is more dynamic, it becomes considerably more challenging for potential adversaries to trace back the origin of a packet. Thus, the safety period of the network is significantly prolonged, too.

The suggested method achieves consistent privacy, independent of the location of the source sensor in the network, while maximizing both the safety period and the network’s longevity. Analytical models are also developed to estimate the average delay the packets take to reach the BS. These models are then used to compare the delay that is obtained through simulations. The details of the proposed protocol are given in Section 4. Additionally, this work studies the impact of sensor node radio range on privacy level and network lifetime, which has not been investigated to date.

The proposed solution (SLP-E) is suitable for deployment in a wide range of natural habitats for wildlife monitoring, particularly in areas where internet access is challenging. In this deployment, the sensor nodes communicate via multi-hop techniques, facilitating the exchange of information packets as they traverse multiple nodes to reach the central sink or base station (BS). In this specific context, it is essential to employ radio frequency identifier (RFID) technology to tag the animals under observation. Each sensor node within the network can be outfitted with an RFID reader, allowing them to effectively detect the assets carrying these RFID tags. Subsequently, the gathered information can be transmitted from the base station to IoT devices connected to the internet for more in-depth analysis. Specifically, this work addresses and mitigates issues associated with cyber-poaching [32], such as resourceful smart hunters equipped with advanced internet-related devices, capable of detecting incoming signals, who may attempt to locate the source of these incoming signals by using a backtracking method that commences at the base station (BS). Another common significant challenge that this work tries to handle is related to the potential degradation of network lifetime seen in existing SLP techniques. Wireless sensor networks typically rely on battery power, and the introduction of privacy measures can lead to increased energy consumption. Striking a delicate balance between the imperative for source location privacy and the critical necessity of prolonging the network’s operational lifespan proves to be a challenging task, particularly within resource-constrained environments. The proposed protocol (SLP-E) is designed carefully by taking into account various tactics that can be employed by a backtracking passive attacker who tries to uncover the origin of data packets and by trying to balance the energy consumption used by each node in the network with the aim of enhancing NLT.

Generally, SLP techniques can be implemented in real-world scenarios for wireless sensor networks; however, they can present several key challenges. These include the complexity of key management in large dynamic networks [33], the delicate balance required between maintaining location accuracy essential for WSN applications and preserving source privacy [34], the added complexity of dynamic network topologies due to sensor mobility and potential failures [24], etc. Addressing these challenges necessitates advanced solutions that can effectively harmonize data accuracy, security, and adaptability. In SLP-E, there are different assumptions made and potential scopes considered. The comprehensive information about the considered assumptions is given in the network model and attacker (hunter) models that have been considered in this study (see Section 3), and the details of the proposed protocol are given in Section 4.

### Contributions of the Work

The main research contributions are listed here as follows:

- Introducing the novel SLP technique (SLP-E), which offers a comprehensive solution by simultaneously enhancing both the safety period and network lifetime. The solution represents a significant enhancement in safeguarding the privacy of source nodes when compared to recent random walk-based SLP solutions. Notably, privacy is enhanced with the improvement in the network lifetime. This is important because sensor nodes are often deployed in vast, challenging environments, making the replacement or tracking of dead nodes a difficult task. Therefore, in addition to ensuring the privacy of the information’s source, there is a crucial focus on extending the network’s lifetime. Moreover, the proposed enhancements are not constrained by the event’s location within the network. Regardless of where an event (animal under observation) appears in the network, the improvements in both the safety period and the network lifetime persist, offering a robust and adaptable solution.
- Developing an analytical model that estimates the average delay of the proposed solution. The work analytically estimates the average packet transmission latency from the source node to the base station in different network settings. We also provide rigorous analyses on privacy through a probabilistic approach.
- Investigating the impact of the radio range of sensor nodes on the network privacy and NLT. This serves to reveal that the transmission range (sensing range) of sensor nodes can either have an impact on or remain independent of both privacy and the network lifetime.

This article’s remaining sections are organized as follows: We present the associated studies in Section 2. We go over the adversary and network models that were taken into consideration in our work in Section 3. Section 4 provides a description of the suggested routing protocol and Section 5 presents the privacy analysis, while the performance measures employed in this work and complexity analysis are presented in Section 6 and Section 7, respectively. The results and comments, as well as the work’s conclusions, are provided in Section 8 and Section 9, respectively.

## 2. Literature Review

Researchers have put forth a variety of routing strategies to address the WSN privacy issue with source nodes. In [15,19,22,25,26,27,35,36], fake packet-based SLP techniques were put out. Since we realized that for energy-constrained WSNs, such schemes (fake packet/fake sources-based SLP) would be energy-expensive, we do not focus on them. Here, we describe only random walk- or phantom routing-based techniques since the proposed scheme falls under this category, as it has been noticed that those techniques are more effective for energy-constrained WSNs [18,21,28,37]. Conti et al. in [23] provide an in-depth review of the source location privacy (SLP) preservation methods suggested for WSN; for more details on this topic, we recommend reading this article. The fundamental goal of employing random walk-based approaches is to make a packet’s journey appear entirely random to an adversary in order to defend against hop-by-hop and traffic analysis attacks. As relaying or forwarding nodes are picked at random from a sending node’s neighbor set, the packet’s journey takes on a random pattern [38].

For the first time, a random walk-based SLP solution was proposed by Ozturk et al. in [38]. Based on the panda hunter game as the baseline, in order to provide SLP in a WSN, their work introduced the phantom flooding scheme (PFS), which is based on conventional random walks. Every message goes through two phases such as a directed walk phase, followed by a flooding phase that sends the packet to the BS. In the directed walk phase, the packet is relayed for up to H hops in a random manner. Baseline flooding is used to flood the data packet once the hop count H reaches zero. The phantom node (PN) is the node at which the hop count H is zero. This solution showed some degree of privacy. Nevertheless, due to the uneven distribution of packet forwarding probability among neighboring nodes, the attainment of a purely random walk was unsuccessful, resulting in compromised privacy. Additionally, the study failed to consider the improvement in the network lifetime (NLT).

Different routing techniques were proposed to improve PFS, including [39,40,41]. In [40], the authors proposed a new SLP routing technique, namely, phantom routing with a locational angle (PRLA). The key idea is to forward a packet to the neighbor node that has the greatest inclination angle with reference to the base station. Each node in the network determines the angle of inclination between itself and its neighbors with respect to the base station. Each node then calculates the forwarding probability using that inclination angle. The chance is greatest for the node with the highest angle of inclination. When an event is detected, a node chooses its neighbor with the highest inclination angle and sends the packet to that node. The above procedure continues until the hop count H reaches zero, or if a node cannot transfer the packet to a neighbor node with the required inclination angle, it converts into a phantom node and forwards the packet via shortest path routing to reach the BS. It was observed that this scheme has an enhanced safety period compared to PSF. This is due to the fact that in PSF, tracing back the source of information can be easy since the packet forwarding probability is less evenly distributed among the neighboring nodes of the source of information, as shown in [41,42,43]. However, the routing path in this scheme lacks the requisite level of randomness necessary to ensure robust privacy for the source of information, and it also neglects the crucial factor of assessing and improving the network’s lifetime.

Li and Ren developed a scheme that uses a three-phase routing strategy [44]. In the first phase, the data packet is sent by the message source to the randomly selected intermediate node (RRIN) in the sensor domain, which subsequently directs it to a ring node. This stage’s goal was to offer local source location privacy. It was projected that the intermediate node would be far from the actual source node, making it difficult for the attackers to learn about the real source from the intermediate node chosen. To provide source location privacy at the network (global) level, the information in the packet is subsequently combined with that of other packets using a network mixing ring (NMR) in the following routing phase. In the end, selected nodes on the mixing ring forward the data packet to the SINK node. This scheme shows the same latency and power consumption as PFS, but its privacy level is higher. Additional routing strategies were proposed by Li and Ren [45,46] to improve this routing scheme for enhancing privacy.

More random walk-based SLP solutions have been proposed by different researchers with the aim of improving existing ones in terms of privacy. To safeguard both the source node and the sink, a source location privacy (SLP) mechanism was created by Chen et al. in [25]. Even though the article proposed four solutions, we consider only the forward random walk (FRW) strategy since the other three strategies are based on fake packets or fake sources, which belong to the different categories of SLP-preserving approaches we consider in this work. Each node in the forward random walk technique randomly chooses a neighbor node that is nearer to the BS when relaying a packet. This guarantees packet convergence at the BS. Since every packet’s destination is the BS, the nodes located in its sensing range send packets straight there. A forward random walk appears to provide a certain degree of privacy when the source node is situated far from the base station. However, significant privacy concerns emerge when the source node is in close proximity to the BS (base station).

For improving existing random walk-based routing protocols, directed random walk was proposed by Gu et al. in [47]. In a directed random walk, each node splits its neighbors into two groups that are opposite one another. Instead of employing a pure random walk, the next hop is chosen randomly from two groups to reach an intermediate node. Then, from the intermediate node, the next hop is chosen from the opposite group. It seems that the suggested scheme may offer some enhancements in the security period. However, the routing path lacks the necessary diversity to confound potential adversaries, and there is a notable absence of consideration for the network’s overall lifetime.

The research in [28] segregates the network into sectors. The selection of the sector is made randomly for each new packet that is sent from the source node. Once the packets reach that intended sector, they travel toward the BS. Sectors that are closest to the source are given lower priority than those that are away from the source. However, this technique exhibits weaker privacy due to its inadequately varied and unpredictable routing path, which fails to effectively mislead potential adversaries. Furthermore, the relationship between privacy and network lifespan has not been investigated.

Lilian et al. proposed another routing scheme in [18] that divides a network into quadrants with the aim of hiding the real source of information from a local adversary. When an asset is detected, a source node sends a packet at random to a predetermined proxy node that is chosen and situated in the quadrant next to its own. The proxy node then uses a forward random walk to deliver the packet to the BS. As the predefined proxy nodes in this work are found in only two quadrants, it may be easier for an adversary to find them as the packets are coming from only those quadrants. Furthermore, since the same set of nodes (from those quadrants) are utilized to deliver the packets to the BS, the NLT may also be impacted.

The article [29] developed a phantom node-based scheme. In this article, the source node chooses a limited number of network nodes to behave as phantom nodes that are not inside a circle encircling the source node whenever an asset is detected. The packets are then transferred via shortest path routing from the source node to the phantom nodes. From there, they are sent in either a clockwise or counterclockwise direction to reach the ring nodes and finally to the BS. In contrast to existing methods, this approach attains the highest degree of anonymity. Nonetheless, it is worth noting that the utilization of the same set of intermediate nodes for packet transmission may have implications for the network lifetime (NLT).

In [34], two solutions with the names PRBRW and PRLPRW were put forth. In PRBRW, packets are sent oppositely initially, and then the greedy technique is used to get to the BS. The limitation of this approach is that there is no improvement in network lifespan. To deal with this limitation, a second strategy, namely, PRLPRW, was suggested. In this technique, the packets are relayed randomly initially and then sent to the BS using min-hop routing. The random walk consists of a vertically up-or-down walk followed by a horizontally left-or-right walk. These two phases sought to increase traffic uncertainty and diversify routing paths. However, the equilibrium between privacy, i.e., the safety period, and the network lifetime problem was not solved using these techniques.

Li et al. proposed a new SLP scheme in [30] to enhance the privacy level of the source of information. In their scheme, the source node sets two candidate domains, and the proxy node that will serve as the source node is chosen from one of the candidate domains. Then, min-hop routing is used by the proxy node to forward the packets to the base station. Considering that the proxy node must be located within one of the candidate domains, all of which are established in close proximity to the source node in the same general direction, there is a potential vulnerability for the attack to discern the source node’s location. This vulnerability arises from the lack of diversity in the routing path. Notably, there was no effort to enhance the network lifetime (NLT), despite the fact that the next hop for packet relay was chosen based on the remaining residual energy.

The authors of [21] proposed an SLP scheme with the aim of protecting path location privacy and congestion avoidance by employing a jellyfish structure. Also, a network is divided into various subdivisions, and sensor nodes placed in these subdivisions select the target area by computing the transmission distance. A virtual ring and radial line are used to protect the routing path from a particular node to the sink, and congestion is prevented by the proposed alternate path routing. To streamline the communication channel between nodes and the sink, the network’s configuration involves the simultaneous selection of the number of radial lines and the virtual ring radius. In this arrangement, bell nodes within the virtual ring are routed probabilistically with varying angular orientations, whereas radial line pathways maintain a fixed routing direction. While this setup appears to offer a notable level of privacy, the recurrent use of the same radial line pathways for packet transmission to the sink raises concerns about potential implications for the network lifetime (NLT).

A bidirectional location-based SLP scheme was proposed in [20] by Zhou et al. for protecting both sink and source node. The scheme’s objectives include improving source and sink node privacy and balancing the quality of end-to-end communication. However, the network lifetime was not taken into account by the authors. An enhanced solution, namely, PSSLP, was proposed in [16]. The PSSLP technique aims to achieve uniform privacy. The notion of dividing a network into different sections was adopted so that the packets, based on distinct phases, are transmitted to the base station. This protocol excels at elevating the privacy of the source node when compared to existing ones. However, it encounters a challenge regarding network lifetime (NLT) due to the utilization of a constant ring, which was intended to enhance routing path diversity, inadvertently resulting in reduced NLT.

It is observed that the majority of these existing techniques do not prioritize the simultaneous improvement of both the safety period and network lifetime (NLT). Although these protocols exhibit a certain degree of improved privacy, they still show some degree of privacy vulnerabilities. Notably, there are shortcomings in terms of routing path randomization and diversity. Furthermore, it is evident that the majority of these solutions aimed at enhancing privacy did not consider the specific position of the source node within the network; consequently, this results in a privacy model that is depending on the source node’s position within the network. The proposed scheme (SLP-E) utilizes a reverse random walk, followed by the walk on annular rings to establish a completely random and diverse routing path to confuse passive attackers. It then employs min-hop routing in conjunction with the walk on dynamic rings to relay the packets to the base station (BS). The routing path in each phase is strategically designed with the aim of enhancing both privacy and network lifetime (NLT) while simultaneously achieving uniform privacy across the network. Furthermore, there has been no research aimed at SLP which investigates the impact of sensor nodes’ radio range on SLP schemes’ important performance metrics. These performance metrics typically encompass the safety period, network lifetime (NLT), entropy, capture percentage, energy consumption, and delay.

## 3. Network and Adversary Models

We describe the network model and adversary model in this section.

### 3.1. Network Model

**Figure 1:**
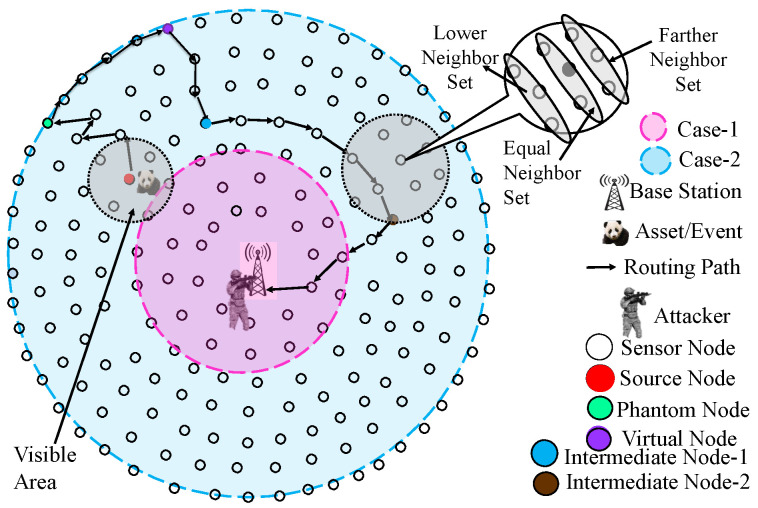
SLP-E technique.

In this section, we present the network model used in this work. This model is inspired by the panda hunter game model suggested in [39]. With a single strong BS in the middle, the sensor nodes are distributed uniformly throughout the network. The network under consideration has a circular structure, and it is envisaged that sensors form rings around the base station, as illustrated in Figure 1. The sensor nodes are uniform and static in design. In other words, before the network starts up, every node in it has the same amount of initial energy, computational power, radio range, and storage capacity. The communication range of the sensor nodes is constrained, so two nodes can only connect when they are close, i.e., within each other’s sensing range. Therefore, multi-hop routing is used for communication between nodes that are not neighbors. Each sensor can determine its position in the network using the existing localization techniques [48]. Here, we take into account the situation of habitat monitoring, in which a network asset could randomly arrive at any point in the network. The asset is assumed to be tagged with an RFID (radio frequency identifier) and sensor nodes are equipped with an RFID reader. A source node continues transmitting the packets to its neighbors after spotting an asset within its radio range to send information packets to the base station. It is expected that the packets have been encrypted using cryptographic methods to prevent unwanted parties from reading their contents [49]. Each node divides its neighbors into three groups: (i) lower neighbor set, (ii) equal neighbor set, and (iii) far-away neighbor set, based on the depth of each node as measured with respect to BS, after the initialization phase (see Section 4).

### 3.2. Attacker Model

The attacker model consists of a local and passive opponent that goes backtracking step by step to identify the true source of the information [25,28,38]. Due to the known position of the BS in the network, and to the fact that all data from the sensor nodes are transferred there, the adversary begins its backtracking operation from the BS. The attacker is assumed to be equipped with tools like computers and spectrum analyzers. The passive attacker performs only eavesdropping and backtracking attacks. Further, it is assumed that the attacker’s device has the same radio range as that of the sensors’ radio range. Before determining the location of the sending node, using its gadgets, the attacker only assesses the message’s signal strength and arrival angle. An attacker goes in the direction of the sender after receiving a packet to determine its location and continues to do it until it finds the packet’s origin.

## 4. The Proposed Method: SLP-E

Here, we go over the specifics of the proposed SLP-E routing method. The two phases that make up the suggested technique are the network configuration phase and the operational phase. The base station invokes the configuration phase so that each node in the network can determine its own position with respect to the base station and also know its neighbors. The SLP-E protocol is executed during the execution phase.

### 4.1. Network Configuration Phase

This work refers to the network configuration process followed in the existing similar random walk-based approaches [28,29,37]. The base station floods the network with a depth configuration message during initialization. The base station’s neighbors are the first ones to receive the depth configuration message. The *depth* field in this message has an initial value of zero. The nodes that receive this packet increase the depth value by one and change the value in their routing table. The packet is then rebroadcast into the network. The nodes that receive these rebroadcast packets add one to the packet’s content, update their respective depth value in their data table, and rebroadcast the packet. This procedure continues until the packet arrives at the network edge. Each node receives information about its one-hop neighbors during this procedure. The neighbor of each sensor node is then divided into three categories: lower neighbors, equal neighbors, and distant or far-away neighbors. An adjacent node is referred to as a lower neighbor if its hop count, also known as the depth value, with respect to the base station is less than the node of interest, an equal neighbor if its hop count towards the base station corresponds to its own, and a distant neighbor if its hop count to the base station is higher. These details are used to route data packets coming from the source node. *Depth* refers to the distance between a sensor node and the BS measured in terms of hops.

### 4.2. Protocol Execution Phase

The actual protocol realization is covered in this phase. We consider two scenarios, namely, scenario-1 and scenario-2. If the source node is at a distance of one-fourth of the network radius as measured from the BS’s position, it is considered as scenario-1; else, it is scenario-2. Since the nodes within the base station’s range are most susceptible to an adversary who initiates the backtracking process from this location, this threshold value is set to apply particular security precautions to those nodes. In Figure 1, the pink color region represents scenario-1 and the blue color region represents scenario-2 of the network.

Depending on where the source node’s position (i.e., its position is in scenario-1 or scenario-2) is in the network, the SLP-E protocol adapts accordingly. This process improves privacy and assists in finding a balance between privacy and the network lifespan by making routing patterns more dynamic. The source node can therefore maintain its anonymity anywhere inside the network.

#### 4.2.1. Scenario-1

A sensor node changes into a source node after detecting an asset and begins transmitting packets to the base station using the proposed SLP-E technique. The following five phases of the routing protocol in this instance are described as follows:

- *Phase-1*: To generate a reverse random walk named a backward random walk (BRW), upon detecting an asset within its sensing range, a source node conducts a check of its location in relation to the base station (BS). If the distance between the source node and the BS is determined to be less than or equal to one-fourth of the network radius measured from the BS’s position, it identifies itself as being in scenario-1. Subsequently, the source node examines the details of its neighboring nodes, which are categorized into lower neighbors, equal neighbors, and distant or far-away neighbors (the specifics of these neighbor nodes are provided in Section 4.1). From this analysis, the source node establishes the farther neighbors as its forwarding set, and among these farther neighbors, it selects the node with the highest energy to forward the packet. The packets are sent in the opposite direction using the farther neighbor set, away from the base station position. The source determines each packet’s hop count to travel in this direction, and this value is chosen randomly between one-third and two-thirds of the network radius (both these values are measured in terms of hops; mathematical analysis that shows the way of calculating the number of hops in each phase is delivered in Section 6.1). We choose these restrictions so that the packet can only travel a certain range (measured in hops). By doing this, when the source node is close to the base station, the packet is prevented from reaching the network boundary. No matter where the source node is in the network, this results in energy conservation, packet delay reduction, and the balance of network lifetime (NLT) and safety period. The hop count number is reduced by one count once this packet is received by a neighbor node, and the neighbor node with the highest residual energy is the one chosen from the list of neighbors to relay this packet. This process continues as long as the hop count value in the packet is non-zero. When the hop count value in the packet is zero, phase-2 of the routing process begins. The phantom node (PN) is the node at which the BRW terminates.
- *Phase-2*: In this phase, the packets are relayed in either a clockwise or anti-clockwise direction. The phantom node decides whether to send the packets in an anti-clockwise or clockwise direction with a coin toss experiment and relays the packets to a neighbor that is in an equal neighbor set. This procedure of transmitting is repeated for *H* hops, where *H* is a random number selected between $45^{\circ }/\theta$ and $180^{\circ }/\theta$, where θ is the hop size in an annular walk and its corresponding number is determined utilizing Equation (4). The virtual source (VS) refers to the node where the transmission process ends in this phase.
- *Phase-3*: From the virtual source, the packets travel in an inward direction towards the BS for certain hops. In this phase, the packets are forwarded to at least two hops in the inward direction from the VS, and the maximum distance they travel is up to the boundary of the inner circle (see Figure 1). The inward walk lower limit in this phase is two hops from the VS, and the upper limit is the boundary of the inner circle (with pink color). Intermediate node-1 (IN1) is found randomly between those set limits. Unlike in the existing solutions where packets are sent directly to the BS through min-hop routing (shortest path routing), in this phase, one additional intermediate node (IN1) is chosen randomly between two nodes away from the VS and the boundary of the inner circle which is showing the threshold value. This improves the routing path’s randomness and diversity. The hop count values used in this phase are taken into account to reduce the likelihood of selecting IN1 which is close to the actual source node. Since in this scenario, a source node is located in the inner circle (threshold circle), we make sure that the intermediate node (IN1) cannot be found inside that circle.
- *Phase-4*: In this phase, the packets consider the direction they followed in phase-2. The packets travel in either a clockwise or anti-clockwise direction to reach intermediate node-2 (IN2). IN1 has to relay the packets in either a clockwise or anti-clockwise direction, as was determined in phase-2. That is, if in phase-2, the packets travel in a clockwise direction, then in phase-4 also the packets will travel in a clockwise direction. The goal of this phase is to reduce the likelihood that a packet will enter the sensing range of the actual source node on its way to reach the BS. If, by any chance, the source node is found at the boundary of the inner circle, as the maximum distance a packet can travel in phase-2 is $180^{\circ }$, if this distance was traveled in a clockwise direction, then in this phase, the packet takes a clockwise direction with $90^{\circ }$ as the maximum distance; with these conditions, the packet cannot visit the source node location. We set $90^{\circ }$ as the maximum distance in order to avoid that a packet may travel a distance which is equal to a complete circle, because doing so when a source node is found at that inner circle boundary means that the packet may visit its place, thus the weaker privacy. This is even the reason why the packets must move in the same direction as they did in phase-2, to avoid the possibility of discovering IN2 within the range of the source node’s radio or passing through it.
  Therefore, in this phase, the packet travels for certain hops whose values are selected between $45^{\circ }$ and $90^{\circ }$. Intermediate node-2 (IN2) is the node where this phase ends.
- *Phase-5*: Finally, by utilizing the shortest path walk, the packet is delivered to the BS from intermediate node-2.

#### 4.2.2. Scenario-2

The routing in scenario-2 is similar to the one suggested for scenario-1 with the following differences: (i) in phase-1, the intermediate node is randomly chosen between $R/2\;\times \;r_{s}$ and the network boundary (measured in hops), and (ii) in phase-3, the hop count value is randomly selected between 4 hops from the VS and $2R/3\;\times \;r_{s}$ in the inward direction, where $r_{s}$ is the sensor node radio range. If the source node is found at the network boundary, then, there is no backward direction; only four phases are to be followed. Due to traffic diversification and energy savings, this aids in enhancing not only the network lifespan metrics but also the safety period, as all nodes in the network participate in routing the packets to the BS in different rounds. This study ensures that all nodes in the network engage in sending the packets to the BS in the different rounds in order to improve NLT, unlike existing solutions where fixed rings are utilized to make the routing diverse or to make shortest path routing route the packets to the BS. The SLP-E proposal is presented in Algorithm 1.
**Algorithm**  **1:** SLP-E
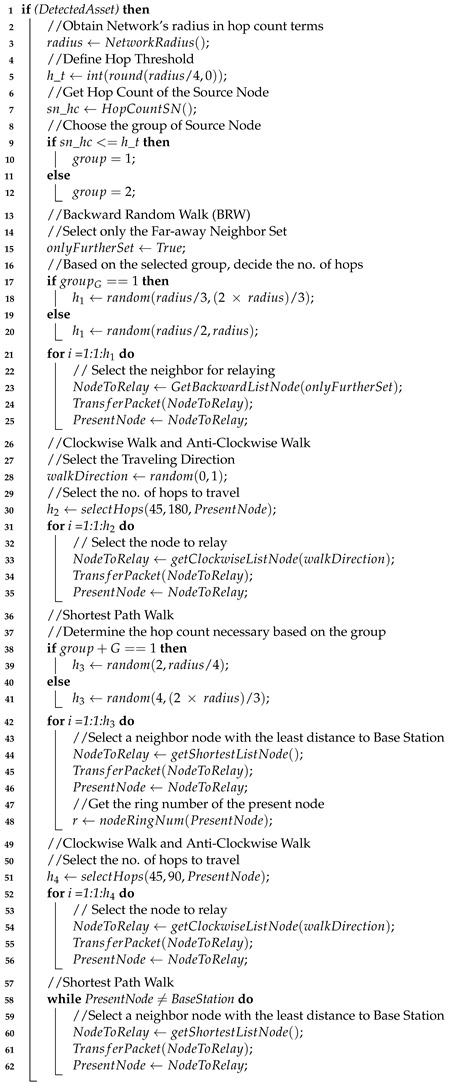


**Figure 2:**
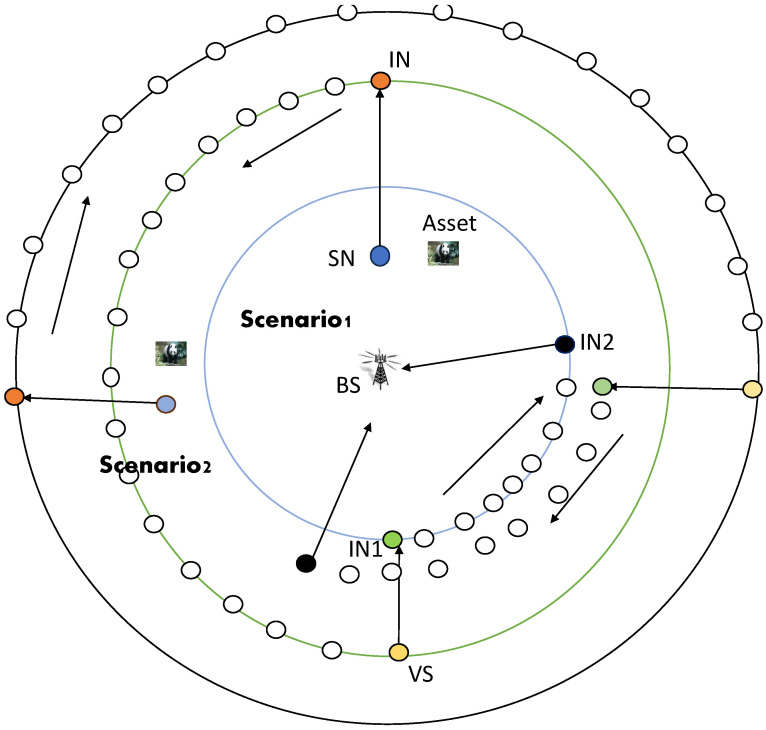
SLP-E routing summary.

Figure 2 summarizes the proposed protocol execution phase and shows how it enhances privacy. Only the upper limits for selecting the intermediate node (IN), virtual source (VS), and IN1 and IN2 in each phase of the two scenarios are shown in the figure. It can be noticed that in both cases, there is no chance for a packet to travel through or close to the source node (SN)’s sensing range on its way to the base station. This is because the direction in which IN2 in phase-4 is picked is the same as the direction in which VS was chosen in phase-2, and that only $90^{\circ }$ distance is taken into account as the upper limit in phase-4, preventing a packet from traveling a full circle distance since $180^{\circ }$ distance is taken into account in phase-2. Phase-1 scenario-1’s upper limit is set at 2R/3rs to prohibit packets from reaching the network boundary once the SN is situated nearby the BS. No matter where the source node is located in the network (close to or distant from the BS), we establish the various restrictions in each phase to balance the hops a packet may traverse on its way to the BS. This helps achieve uniform privacy, prevents unnecessary delays, and reduces energy use. The adoption of multiple phases and the setting of lower and upper limitations are conducted in order to increase the randomness of the routing path and try to use every sensor node in the network to send packets to the BS; this balances energy consumption of all the sensor nodes in the network and aids in improving the NLT.

## 5. Privacy Analysis

**Figure 3:**
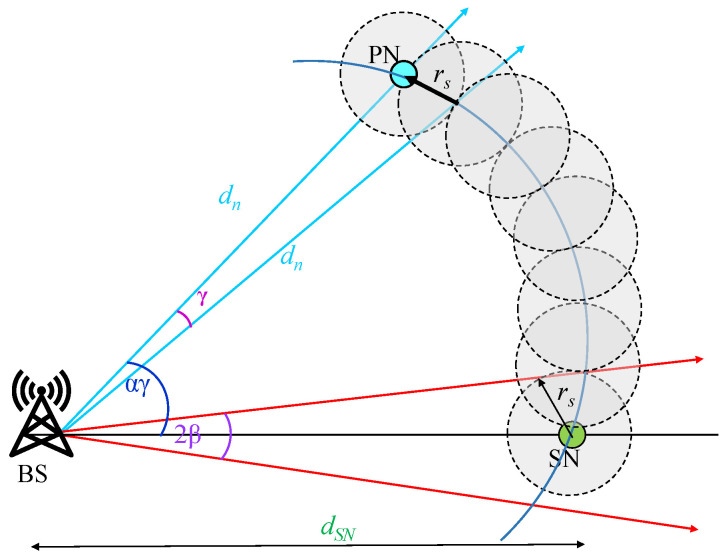
Privacy analysis.

*Privacy analyses* in our work indicate the packet escape probability $p_{esc}$ from the attacker’s sight. Figure 3 shows this effect. Let us investigate a source that does not use SLP and sends a lot of packets via shortest path routing. In that scenario, every packet will arrive at the base station (BS) within the inference space that is determined by the angle 2β. The reason is that the SN packets travel a single path to reach the BS. This helps the attacker to reach the SN within $d_{SN}/r_{s}$ hops, where $d_{SN}$ is the distance between the BS and the SN. The two red-colored arrows drawn tangent to the SN indicate the total angular space (2β) occupied by the SN compared to the entire network angle 2π. Any path trajectory of the packets that escape this region is never intercepted by this region (2β). To mitigate the issue of this direction-based inference attack by the attacker, it is suggested that the packets escape this visibility space. The space within the angle (2β) made by the SN is termed as the *visibility space*. In order to accomplish this, as illustrated in Figure 3, the random walk-based SLP-E protocol seeks to transmit the packets far from the source node to a distant node known as the *phantom node* (PN). From here, the packets are sent to the BS using annular walks. That means if the packets have to escape the SN’s vicinity, then they have to travel for a distance of αγ (measured in hops) from the SN point. Precisely, we use and suggest packet escape probability $P_{esc}$ to measure the percentage of packets that escape the SN visibility space which is given by

(1) $$P_{esc}=1-\frac{\beta }{\alpha \gamma }$$

where β is $sin^{-1}\left(\frac{r_{s}}{d_{CN}}\right)$, α is number of hops towards the phantom node, and γ is $cos^{-1}\left(\frac{2d_{n}^{2}-2r_{s}^{2}}{2d_{n}^{2}}\right)$. The ratio $\frac{\beta }{\alpha \gamma }$ indicates the probability of packets being intercepted by the attacker if it is in the visibility space. If the term αγ is larger than β, then packet interception probability is low; otherwise, it is one, when αγ is smaller than or equal to β. Since the proposed scheme uses divergent routes to send the packets to the BS, the majority of the packets travel beyond the visibility space and thus provide better privacy.

## 6. Performance Characterization

The proposed SLP-E protocol is evaluated based on the metrics listed below:

1. *Safety period*: it is described as the total number of packets sent to the BS until the adversary finds the actual information source [38].
2. *Capture percentage*: the capture percentage is calculated as the fraction of trials in which an attacker successfully reaches the source node over all trials in the simulations.
3. *Entropy*: We quantify the randomness of the routing paths using this metric. With higher entropy, the enemy is more confused, hence the prolonged safe period. This measure is provided by
  $$\begin{array}{c} H\left(Z\right)=-\sum P\left(X\right)\times log_{2}P\left(X\right), \end{array}$$
  where P(X) represents the probability function for the random variable *X*. Assume that $Y(n_{i})$ represents the number of packets sent by every sensor node ($n_{i}$), whereas $Y_{t}$ represents the total number of packets sent by the source throughout the simulation. Entropy is then provided by
  $$H\left(Z\right)=-\sum_{i=1}^{i=Z}\left(\frac{Y(n_{i})}{Y_{t}}\right)\times log_{2}\left(\frac{Y(n_{i})}{Y_{t}}\right).$$
  The number of nodes in the network is represented by *Z* in this scenario.
4. *Energy consumption*: Based on the energy used to send and receive a message, the energy consumption is calculated. We employ the power consumption methodology recommended in [50] to calculate this measure. The notations that are used to analyze energy consumption are shown in Table 1, and here are the equations:
  $$E_{T_{X}}\left(l,d\right)=E_{elec}*l+e_{fs}*l*d^{2},\;d<=d_{0};$$
  $$E_{T_{X}}\left(l,d\right)=E_{elec}*l+e_{amp}*l*d^{4},\;d>d_{0}.$$
  where the energy consumption for receiving a message of the length *l*-bits is given by:
  $$E_{Rx}\left(l\right)=l*E_{elec}.$$
  where the energy loss, $E_{elec}$, is a function of the transmit circuit’s filtering, signal spreading, and digital coding. The energy of the amplifier, $f_{s}*l*d^{2}$ and $e_{amp}*l*d^{4}$, depends on the receiver’s location and the allowed bit-error rate.
5. *Maximum delay*: We can count the hops that packets must make from their origin to the base station using the delay metrics. In Section 6.1, we come up with the formula for the maximum delay metric.
6. *Network lifespan or lifetime (NLT)*: it is determined by how many packets are sent in the network before the first node runs out of power.

The symbols utilized in this work are displayed in Table 1.

### 6.1. Delay Estimation Models

**Figure 4:**
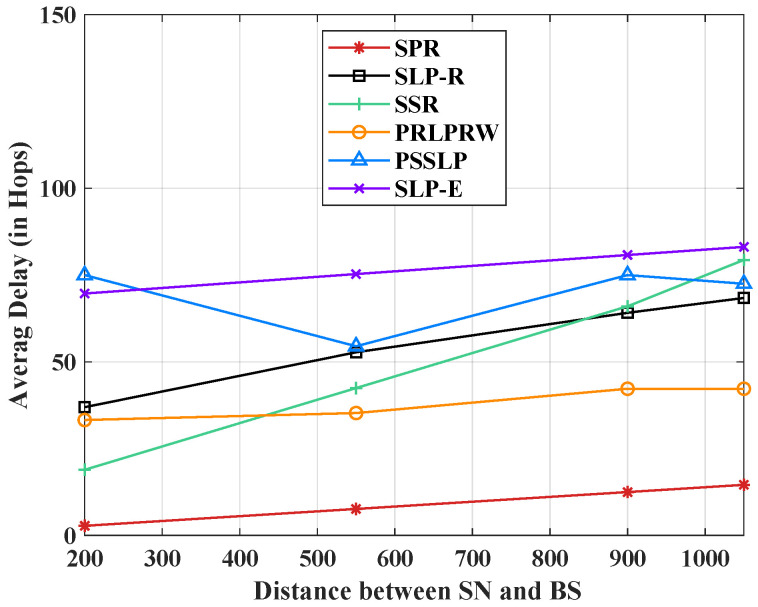
Maximum delay analytically (in hops).

The analytical models for the suggested solution’s *maximum delay* or *hop count* are derived in this section. Plotted in Figure 4 are the delays obtained through the developed model.

#### 6.1.1. Scenario-1

**Figure 5:**
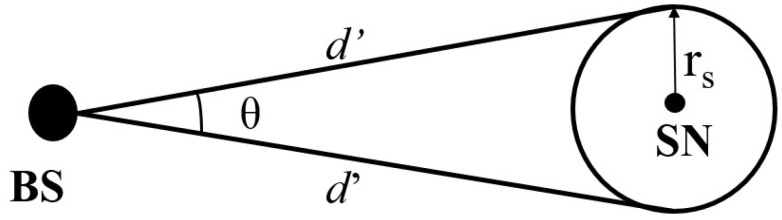
Theta estimation.

If the distance (in hops) between the source location and sink is below the established threshold, SLP-E has five phases, with the following maximum delay for each phase:

- *Max Delay in Phase-1*: There is a backward random walk between R/3 and 2R/3. Let *d* be the distance between the source node and the sink. The BRW packets travel a distance of $\left(\frac{2R}{3}-d\right)$ units. Therefore, the number of hops in *phase-1* is:
  (2) $$H_{11}=\frac{\frac{2R}{3}-d}{r_{s}\times p_{r}},$$
  where $r_{s}$ is the radio range of the sensors and $p_{r}$ is the proportion of neighbors at equal and higher depths to all neighbors.
- *Max Delay in Phase-2*: The farthest distance that may be covered in *phase-2* is $180^{\circ }$; therefore, we have:
  (3) $$H_{12}=\frac{180^{\circ }}{\theta },$$
  where θ is calculated as follows: from Figure 5 and using the cosine angle rule, we have
  (4) $$\begin{array}{cc} \theta & =\cos^{-1}\left(\frac{2{d^{\prime }}^{2}-4r{{}_{s}}^{2}}{2{d^{\prime }}^{2}}\right), \end{array}$$
- In this phase, packets move through a distance to reach a sensor that is at R/4 units as measured from the BS. The maximum distance traveled in this phase is $\frac{2R}{3}-\frac{R}{4}$, i.e., $\frac{5R}{12}$ units. Therefore, the number of hops in this phase is given by
  (5) $$\begin{array}{c} H_{13}=\frac{5R}{12*r_{s}\times p_{r}}, \end{array}$$
- *Max Delay in Phase-4*: The greatest distance covered at this phase is $90^{\circ }$. Consequently, the number of hops in this phase is provided by
  (6) $$H_{14}=\frac{90^{\circ }}{\theta },$$
- *Max Delay in Phase-5*: The distance traversed by the packets in this phase is R/4 units; therefore:
  (7) $$H_{15}=\frac{R}{4rs},$$
  Summing up, we have:
  (8) $$H_{t_{1}}=\frac{\frac{2R}{3}-d}{r_{s}P_{r}}+\frac{180^{\circ }}{\theta }+\frac{2R}{3rs}+\frac{90^{\circ }}{\theta }+\frac{R}{4rs}.$$

#### 6.1.2. Scenario-2

The maximum delay in all five stages of SLP-E is as follows if the distance between the source site and sink (measured in hops) is greater than the permitted threshold:

- *Max Delay in Phase-1*: Between R/2 and *R* is the backward random walk. Then, R−d units are offered for BRW. Hence, the maximum number of hops in BRW is given by
  (9) $$H_{21}=\frac{R-d}{r_{s}\times p_{r}},$$
- *Max Delay in Phase-2*: Phase-2’s maximum distance traveled is $180^{\circ }$; hence, the number of hops in this phase is:
  (10) $$H_{22}=\frac{180^{\circ }}{\theta },$$
- *Max Delay in Phase-3*: Here, four hops are the minimum, and 2R/3 hops are the maximum. The distance traveled by packets in phase-2 is at most up to *R*; hence, the maximum delay or hops in this phase is $2R/3r_{s}$, in the inward direction from the network edge.
- *Max Delay in Phase-4*: The maximum length covered in this phase is $90^{\circ }$:
  (11) $$H_{24}=\frac{90^{\circ }}{\theta },$$
- *Max Delay in Phase-5*: The maximum distance we have is *R*, and the remaining path to be traversed by packets in SPR after *phase-4* is $R-\frac{2R}{3}$; therefore, we have:
  (12) $$\begin{array}{c} H_{25}=\frac{R}{3r_{s}},\;\left(in\;hops\right) \end{array}$$
  The total number of hops $H_{t_{2}}$ is
  (13) $$H_{t_{2}}=\left(\frac{R-d}{r_{s}*P_{r}}\right)+\frac{180^{\circ }}{\theta }+\frac{R}{3r_{s}}+\frac{90^{\circ }}{\theta }+\frac{2R}{3r_{s}}.$$

Figure 4 shows the overall routing protocols’ maximum delay, expressed in terms of hops. These are the hops that the packets travel over to reach the BS. When compared to the shortest path routing (SPR), SLP-R [37], PRLPRW [34], SSR [28], and PSSLP [16] schemes, the proposed scheme has the largest delay. Furthermore, the proposed scheme’s delay graph is nearly straight, demonstrating that it balances the number of hops a packet must take to reach the BS at any location of the source node in the network as compared to the existing protocols. Hence, the balanced NLT and safety period.

## 7. SLP-E’s Complexity Analysis

In this section, we provide a comprehensive analysis of the algorithm’s complexity, specifically focusing on the number of message transmissions needed to successfully convey the packet from the source to the base station (BS). The proposed algorithm begins by initializing the network through a flooding message, which is continually rebroadcasted at each node until it reaches the network’s boundaries. In the case of a network consisting of *N* nodes, the initialization phase involves the transmission of messages in the order of O(N). This characteristic holds true for all other existing SLP techniques. During the operational phase of SLP-E, each source node transmits a packet to the base station (BS), and a node that receives this message forwards the packet to its neighbor that lies in the routing path. This entails message transmission of order O(1) at each node per hop. The total number of hops the message traverses in the network depends on the distance between the source node and the BS and the routing protocol design. The message transmission complexity for the routing phase depends on the average number of hops the packets take to reach the BS. This analysis is given in Section 6.1 and Equations (8) and (13). Therefore, the total number of message transactions in the network of the proposed scheme is given by

(14) $$\begin{array}{c} No.ofmessagetransactions=O\left(N\right)+\frac{\frac{2R}{3}-d}{r_{s}P_{r}}+\frac{180^{\circ }}{\theta }+\frac{2R}{3rs}+\frac{90^{\circ }}{\theta },\\ =+\frac{R}{4rs}+\left(\frac{R-d}{r_{s}*P_{r}}\right)+\frac{180^{\circ }}{\theta }+\frac{R}{3r_{s}}+\frac{90^{\circ }}{\theta }+\frac{2R}{3r_{s}}. \end{array}$$

## 8. Results and Discussion

In this section, we go over in great depth the simulation parameters as well as the assessments of the simulation outcomes. To assess our technique’s performance, we first contrast it with shortest path routing (SPR) and then with other similar approaches (i.e., SLP techniques based on random walks). Comparisons are made using the SPR (no privacy strategy), SLP-R [37], SRR [28], PRLPRW [34], and PSSLP [16] schemes. The performance indicators used to assess the efficiency of the suggested remedy are described in Section 6.

### 8.1. Simulation Settings

The following simulation settings and parameters are taken into account. We take into account a circular deployment architecture with a network radius of 1050 units, as shown in Figure 1. Based on the factor $r_{s}\times \sqrt{2}/2$, we take into account an *offset* distance of 50 units between two nodes. *Offset* is defined as the spacing between two adjacent nodes in the network. Except for the boundary nodes, this option guarantees that each node has eight neighbors. Using this number, we calculate the radio range of the sensor to be 72 units, the total number of nodes in the network to be 1460, and the total number of annular rings to be 21. We consider two groups (scenarios) where one group’s nodes are within a distance of R/4 in hops with reference to the BS, and otherwise for the other group. R/4 units are chosen as the threshold value. Here, we consider a single source node and change its location within the network using BS as a point of comparison. We run 100 trials and send 1000 packets to the base station for each position of the source node. Each trial’s simulation comes to an end when an adversary finds the source node or when all packets are transmitted to the BS. The base station, which serves as the hub for all incoming network traffic, is where the enemy begins its backtracking procedure. Each sensor node in the network is initially charged with 0.5 joules of energy. In this example, we use the locations of the source nodes: (200, 0), (550, 0), (900, 0), and (1050, 0). We use the Python programming language to implement and simulate the performance of the proposed technique.

### 8.2. Result Analyses

In this section, we present the results and explain them in detail.

**Figure 6:**
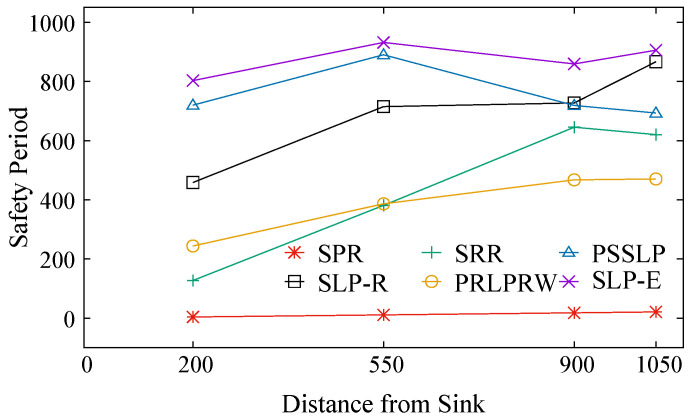
Safety period.

The plots for the safety period metrics are shown in Figure 6. It is observed that the PRLPRW technique performs better when the source is closer to the BS and in the mid-range of the network. However, as the distance between the source and the BS increases, the safety period has a lower value than in all other protocols, except the SPR technique. In SRR protocol, the safety period increases as the distance between the source and the BS increases. This trend is seen for distances up to 900 units. Beyond this distance, the safety period decreases. A similar trend of increasing safety period with the increase in the distance between the BS and the source is seen in the SLP-R technique. In this case, there is an increase in the safety period metric for all distances between the source and the BS. In these three techniques, the safety period is having larger values for the larger distances between the source and the BS. The reason behind this trend is that these protocols have distance-dependent behavior. That is, the larger the distance of separation between the BS and the source node position, the larger the safety period. In the PSSLP technique, the safety period has larger values for all the positions of the source node, except when it is closer to the network edge where its value decreases marginally. In the proposed technique, the safety period metric has the highest value compared to other solutions. This holds true for all source node-to-base station (BS) distances within the network, and it is achieved because we ensured an equitable distribution of the number of hops a packet must traverse before reaching the base station (BS), regardless of the source node’s placement within various network settings. It is seen that the existing protocols have a different trend in safety period metric value when the source is closer to the network edge (i.e., in between 900 and 1050 units of distance from the BS). This phenomenon can be attributed to the fringing effect. However, in the proposed protocol, this is not the issue.

We also observe that SPR has the smallest safety period metric, as it does not focus on privacy. That is, the amount of safety period is minimal when the source is close to the sink. As the distance between the source and the sink grows, so does the safety period. When the source node is close to the BS, PSSLP operates pretty well. However, when the source node is close to the network boundary, the safety period shortens. Thus, present methods have non-uniform safety periods. No matter where the source is in the network, SLP-E has nearly the same level of privacy. This behavior of SLP-E is attributed to the fact that the proposed scheme adapts the routing protocol based on the position of the source in the network, i.e., whether it is scenario-1 or in scenario-2. Thus, the goal of attaining uniform location privacy is accomplished.

**Figure 7:**
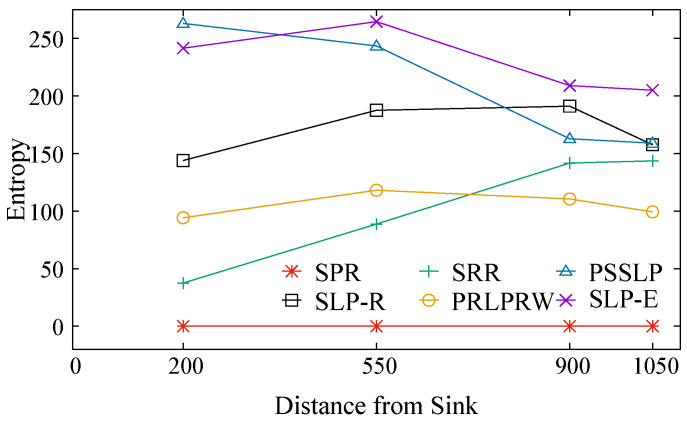
Entropy.

The degree of randomization in the routing path is indicated by the entropy metric. The entropy increases with the degree of randomness in the routing paths that packets take. In comparison to the other five strategies, the suggested SLP-E has the best entropy, as shown in Figure 7. When compared to other protocols, SRR has less entropy. All source position settings in the network exhibit this tendency. Because routing protocols do not take the location of the source in the network into account, PRLPRW, SLP-R, and SRR have low values of entropy. Compared to other protocols, PSSLP has the highest entropy, although its value is lower than that of the suggested approach (SLP-E). This is because PSSLP’s final routing phase is not entirely randomized. As opposed to SLP-E, which completely randomizes all of the routing phases, the source’s position in the network relative to the BS’s position is taken into account when routing decisions are made. When evaluating SLP-E’s entropy in comparison to other network settings, it becomes evident that its performance excels at both the 200 and 550 levels. This distinction is attributed to the fact that in scenario-1 (the source node is nearby the BS), the packets from the source node undergo a fully randomized backward walk towards the intermediate node (IN). This is due to the fact that the IN is chosen from a higher quantity of randomly generated numbers in scenario-1, phase-1 (see Section 4 for details). In contrast, in scenario-2, a source node may be located in proximity to or at the network’s edge. In this case, the selection of an intermediate node (IN) is either non-existent or entails generating only a limited number of random nodes for IN selection. SPR has a defined routing path with no entropy because it does not offer any privacy mechanisms. In all five SLP protocols, we see a somewhat declining trend in entropy when the source is close to the network edge. Due to boundary effects and the network’s geometry, this behavior is expected.

**Figure 8:**
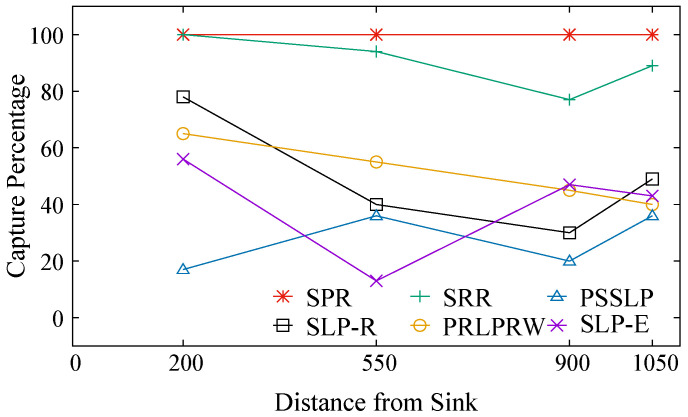
Capture percentage.

The adversary’s success rate is indicated by the metric capture ratio. As a result, the performance of the protocols improves as the magnitude of this parameter decreases. As seen in Figure 8, the suggested scheme’s capture ratio is the lowest when compared to the SLP-R, SRR, and PRLPRW procedures. In contrast to PSSLP, SLP-E has a higher capture ratio. Increased NLT in SLP-E relative to PSSLP makes up for this loss. As the separation between the source and the sink grows, PRLPRW is essentially linear with a small decreasing tendency. As the separation between the source and the sink widens, the capture ratio metrics for SLP-R and SLP-E both show a decreasing trend, followed by an upward trend for the other positions of the source in the network. The capture ratio of SLP-E demonstrates superior performance at the 550 setting in comparison to other configurations. This is a result of our efforts to enhance both NLT and privacy uniformly, accomplished by the implementation of various restrictions, thus resulting in this trend. Moreover, it is evident that, on average, the capture ratio of SLP-E outperforms that of other protocols, except for PSSLP.

**Figure 9:**
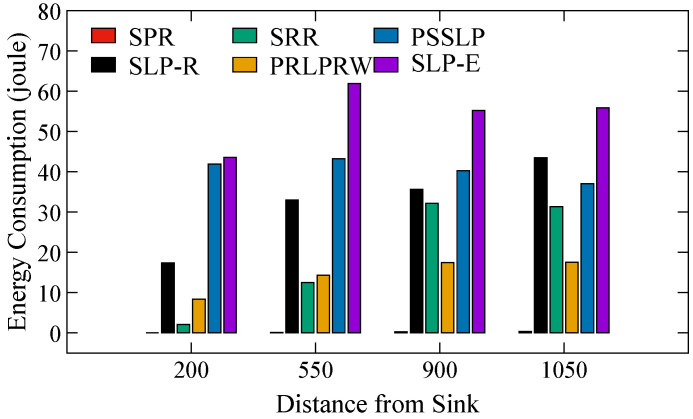
Energy consumption.

Figure 9 displays the energy usage metric charts. Energy usage per packet per hop during packet transmitting and receiving was calculated using the energy models given in Section 6. It demonstrates that, when compared to all other existing schemes, including those without SLP, the proposed design SLP-E consumes the most energy. However, it should be noted that SLP-E uses more energy because it can transmit more packets to the BS than any other method. In comparison to the suggested strategy, the simulation is over faster when the attacker reaches the source in the SLP-R, SRR, PRLPRW, and PSSLP schemes. This confirms that fewer packets are sent in SLP-R, SRR, PRLPRW, and PSSLP, which further suggests that less energy is used. We utilize a different statistic called network lifetime (NLT) to assess the effectiveness of the proposed scheme’s overall energy use. In terms of the network’s lifespan, the NLT metric shows that SLP-E is more energy-efficient than SLP-R, PRLPRW, SRR, and PSSLP. It is observed that in SLP-E, trade-offs exist between energy usage metrics and privacy level. By definition, the energy consumption is the amount of energy used by each network node when sending and receiving a packet. Given that SLP-E’s primary goal is to provide increased privacy in conjunction with the NLT, and given that SLP-E employs the random walk routing technique, it is imperative to offer a more randomized path for a packet to travel from the source node to the base station. In order to make routing path more random and diverse enough to enhance the privacy, the source node must send a significant number of packets to the BS before the adversary locates it, and each packet should visit a significant number of nodes before reaching the BS. Enhancing the randomness and diversity of the routing path in SLP-E improves privacy but results in higher energy consumption when compared to alternative routing protocols that offer lower levels of privacy and randomization. However, even with those other protocols, energy usage is high in comparison to their degree of privacy. The advantage of SLP-E is that, despite using the most energy, practically every network node participates in routing various packets to the base station (BS), which improves NLT. This is in contrast to the other approaches, which use the same sets of nodes to send packets repeatedly, leading to poor NLT.

**Figure 10:**
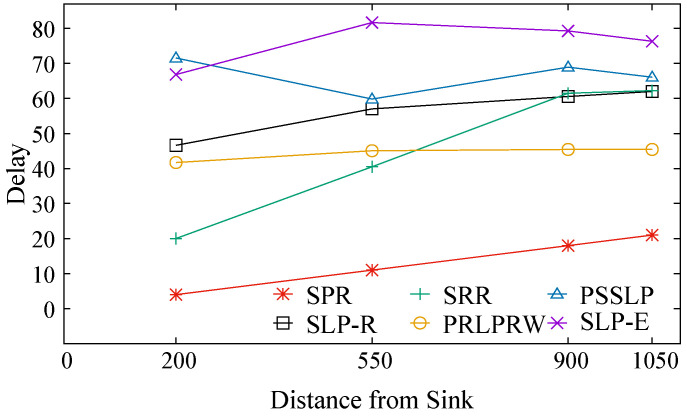
Delay.

We talk about the average delay measure as it is represented graphically in Figure 10. This indicator shows how long it typically takes for packets to arrive at the base station. This metric counts the number of hops a packet must traverse to reach the sink. A hop equals one delay unit. Plots of the delay metrics for five SLP procedures and one non-SLP technique are shown in Figure 10. Compared to all other protocols, SLP-E has the highest delay since it uses longer pathways to improve anonymity. The SPR protocol is the least time-consuming because it offers no privacy protection. Also, the maximum delay estimated using the analytical models and presented in Figure 4 closely matches the trend of the average values of the delay metric present in Figure 10, the simulation values. Hence, both the theoretical and experimental results are consistent with each other. As mentioned earlier, the trade-off observed between packet transmission latency and the level of privacy in SLP-E is because the SLP-E routing protocol sends the packets along a highly random and diverse path to reach their destination (the BS). This trade-off is a common characteristic in most SLP solutions based on random walks.

**Figure 11:**
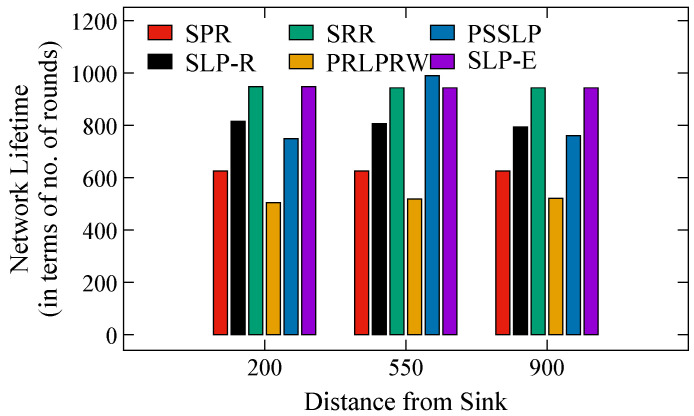
Network lifetime.

Next, we talk about how long the network will last if SLP protocols are used to safeguard the asset. In this scenario, a lot of packets are continuously delivered from the source to the BS until the first node in the network runs out of battery power and is deemed to be dead. The simulation comes to an end once a dead node is found. The number of packets transferred to the BS as a whole is then counted to obtain the network lifespan metric. Figure 11 displays the charts for the network lifespan metric. It can be seen that the proposed scheme performs equally well along with the SRR technique. As discussed earlier, we have demonstrated that the NLT of SLP-E performs better on average. Accordingly, the suggested strategy continues to be the optimum for obtaining a consistent and improved safe period and NLT. This achievement in SLP-E is a result of a well-designed routing protocol, which avoids repeatedly using the same set of nodes to deliver packets to the BS. In addition, each node takes into account the remaining energy of its neighboring nodes when selecting the next relay node in the forwarding process.

### 8.3. Impact of Radio Range on Privacy Strength

In a wireless sensor network (WSN) that relies on multi-hop communication, the sensor node radio range is a critical parameter with several considerations. Selecting the appropriate radio range in a multi-hop communication WSN involves balancing coverage, connectivity, energy efficiency, interference management, security, and other application-specific requirements [51]. Careful design and optimization of the network’s physical and data link layer parameters are essential to ensure the WSN operates effectively and efficiently [52]. Since this work aims at enhancing source location privacy by introducing a new SLP protocol (named SLP-E) to prevent a passive attacker from locating the source node easily, in this study, we exclusively investigate the influence of sensor nodes’ radio range on the performance parameters of SLP solutions.

As it was mentioned in the previous sections of this work and in the existing SLP schemes, it is crucial to make sure that the established SLP schemes should put an emphasis on improving assets’ level of privacy and lengthening the lifetime of the network. However, it is pointed out that there has been no investigation conducted to assess the potential influence of the radio range of the deployed sensor nodes on both privacy levels and the longevity of the network lifetime in the context of SLP.

**Figure 12:**
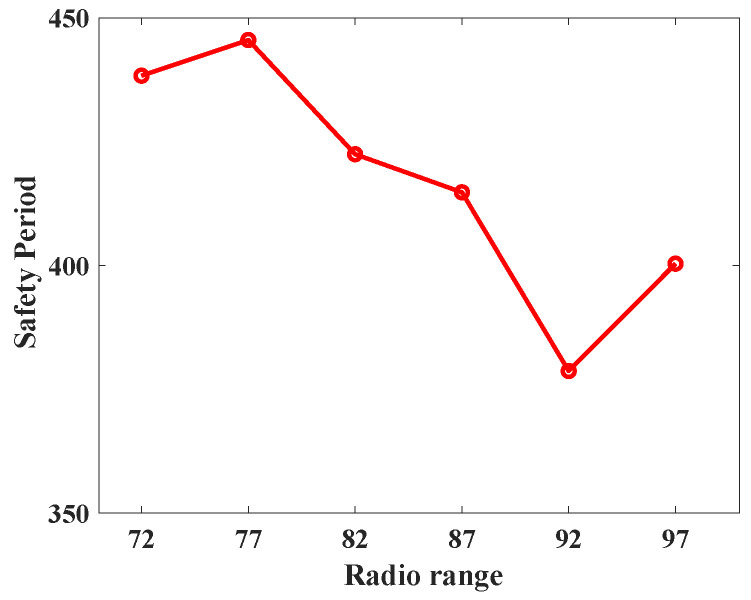
Safety period.

**Figure 13:**
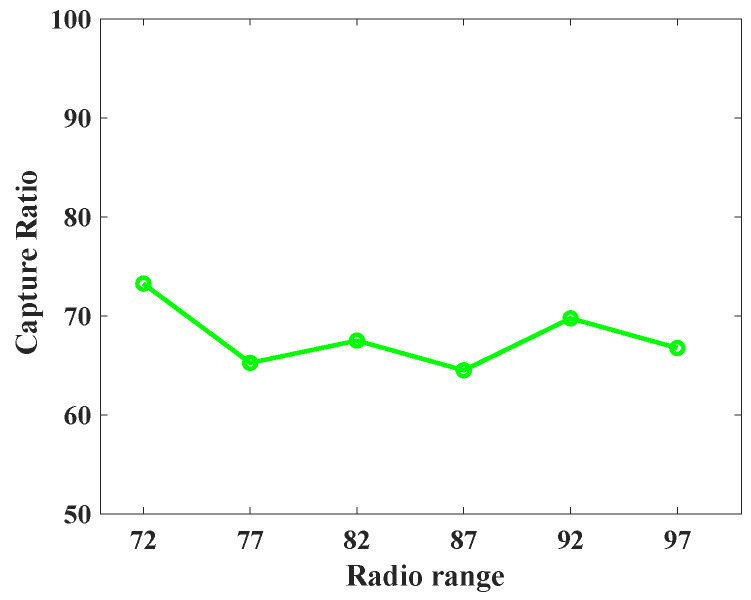
Capture ratio.

**Figure 14:**
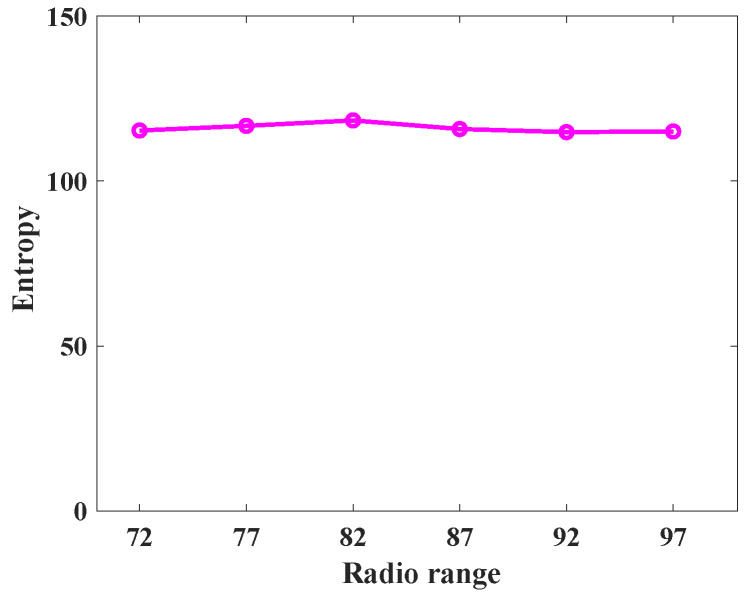
Entropy.

**Figure 15:**
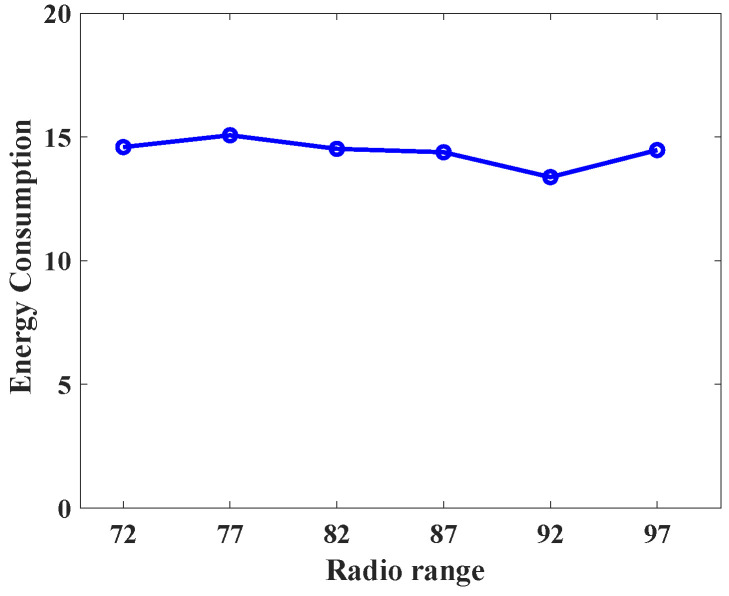
Energy consumption.

**Figure 16:**
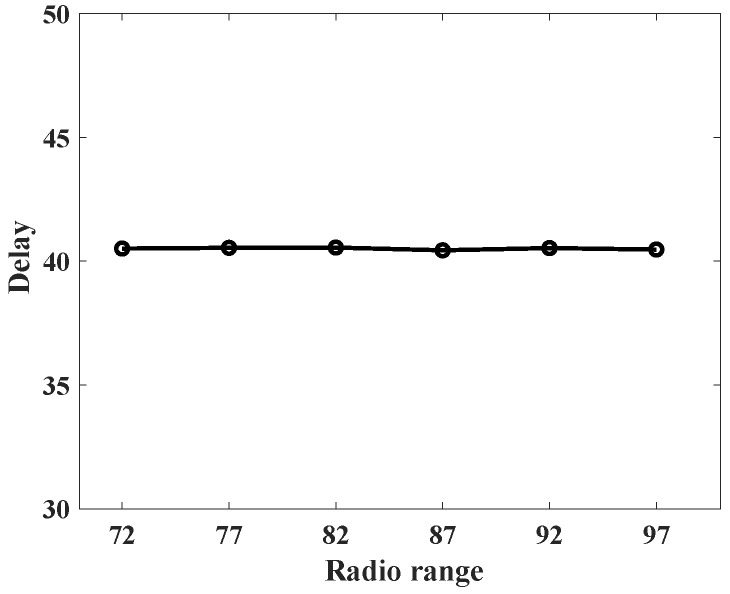
Delay.

**Figure 17:**
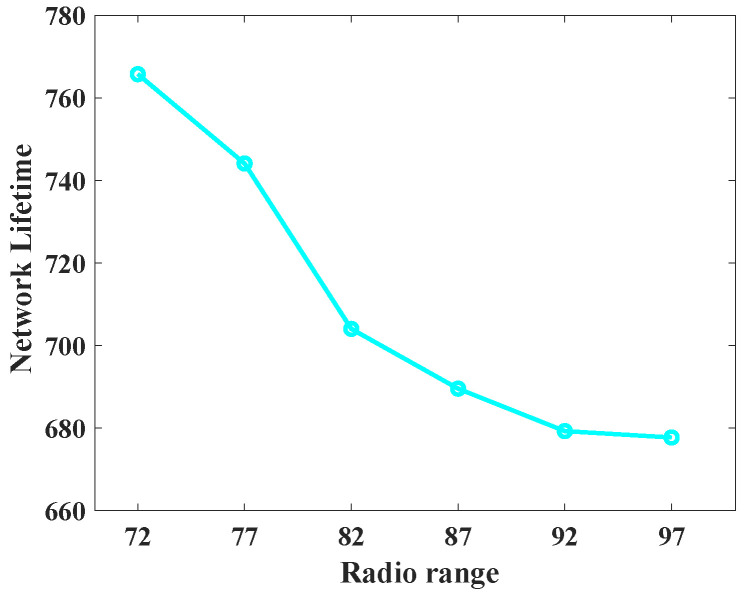
Network lifetime.

To understand the behavior of the proposed protocol (SLP-E) under different radio range settings, we experimented and collected additional results. In particular, the simulation settings for this scenario remained the same as stated in Section 8.1, but this time the sensors’ radio range was varied in a step size of five starting from 72 units. That is, we tested the proposed protocol with the following radio ranges: 72, 77, 82, 87, 92, and 97. The objective of this scenario was to answer our long-awaited query, “will a radio range have any influence on source privacy strength?”, and we try to answer it here. For this scenario, we have taken the average of all the values for different distances between the source and the BS and summarized the results as shown in Figure 12, Figure 13, Figure 14, Figure 15, Figure 16 and Figure 17.

The plot for safety period vs. different values of radio range is shown in Figure 12. It is seen that as the radio range increases, there is a decreasing trend in the safety period for radio range values up to 92 units, except for when the radio range is 77 units. After that, again the safety period sees an increasing trend. The safety period is highest when the radio range is 77 units.

The capture ratio metric vs. radio range plot is seen in Figure 13. This metric has an alternating increase and decrease in the values as the radio range increases. The capture ratio is the least when the radio range is 77 and 87. Figure 12 and Figure 13 indicate that a radio range value of 77 units gives better results in terms of safety period and capture ratio.

Figure 14, Figure 15 and Figure 16 show the plots for entropy, energy consumption, and delay metrics. It is seen that these three metrics almost have the same magnitudes for all the settings of the radio range, with minor deviations in energy consumption. It can be concluded that these three metrics are not much influenced by the radio range of the sensor nodes.

The plot in Figure 17 is for the network lifetime metric. It is evident that the network lifetime metric is largely influenced by the radio range of the sensor node. The inference is that for the given spacing arrangement of the nodes in the network, a smaller radio range has a better network lifetime.

Overall, it is concluded that the radio range of 77 units is doing good in terms of safety period and 72 radio range units is good for capture ratio and network lifetime. It is observed that when the sensor nodes’ radio range increases, the safety period and the network lifetime decrease. Future studies could investigate the cause of this behavior.

### 8.4. Discussions

A list of all metrics that have been averaged over each scenario setting are found in this section. These average values are shown in Table 2. We use shortest path routing (SPR) as our reference methodology to determine the percentage of increase or decrease. The table shows an improvement in SLP performance. The SLP-E’s safety period has grown by 6379 times compared to SPR, while SLP-R, SRR, PRLPRW, and PSSLP have improved 5024-, 3185-, 2803-, and 5479-fold, respectively. It is also seen that both NLT and entropy measurements exhibit comparable increases (see Table 2).

The NLT metric’s negative value shows that PRLPRW performs worse than the SPR (no SLP) scheme. We want to emphasize that the PRLPRW protocol was suggested and evaluated by the authors for multi-asset scenarios. However, in our research, we have only tested this technique for the situation of a single-asset scenario. We stress that the metric capture ratio has a negative value because it reflects the degree of information loss from the viewpoint of the network administrator. The success rate of the attacker is lowered and the SLP protocol’s privacy strength is increased with decreasing values of the capture ratio measure. SLP-E is found to have a lower capture ratio statistic than the SLP-R, SRR, and PRLPRW schemes.

Higher energy usage and delay come at the expense of SLP-E’s increased privacy strength and NLT. In other words, if privacy must be improved, energy usage per packet and delay must be given up. As a result, we discover that the suggested solution uses somewhat more energy and has a little longer delay than any competing schemes. Future efforts could focus on enhancing these metrics.

According to our research, the safety period and network lifespan measures have demonstrated enhanced performances and behavior that is independent of distance. Consequently, the goal of achieving balanced, uniform, and improved privacy and network lifetime measures has been attained. The results of our study suggest that metrics such as entropy and capture ratio continue to behave in a distance-dependent manner. In other words, the values of these two measures vary depending on where the source is located within the network.

### 8.5. Future Research Direction

Based on the findings, we suggest that future research can focus on developing a multi-objective optimization that takes care of sensor spacing, radio range, etc., as optimization parameters to provide an optimal solution. It is strongly believed that q-learning-based approaches can be employed to provide optimal solutions. There are works in the literature that have focused on q-learning-based solutions for routing purposes. However, to the best of our knowledge, there is no work that exists that has explored a reinforcement learning approach for providing SLP solutions. So, there is scope to explore this dimension, too.

Another research direction that can be looked at is the optimal choice for the threshold concept that we used in this work. Although the threshold value of R/4 taken to decide scenario-1 and scenario-2 in the proposed solutions is arbitrary, we were motivated by the fact that nodes near the BS have to expend more energy compared to the nodes that are closer to the network edge. Investigating the ideal size and quantity of these network segments used to improve privacy and security may be another study area. Additionally, future research may focus on more exploring and handling the trade-offs between energy consumption, packet delay metrics, and source location privacy level seen in SLP-E and in other existing SLP solutions.

## 9. Conclusions

In this article, we suggest a novel privacy-preserved routing scheme that enhances privacy and network lifetime for WSNs. The performance of the suggested method shows an improvement in the safety period and network lifespan by 6379% and 50%, respectively, when compared with no privacy routing technique (SPR). When compared to the existing SLP solutions, SLP-E shows improvement in the safety period by 26.5%, 97%, 123%, and 15.7 in the SLP-R, SRR, PRLPRW, and PSSLP techniques, respectively. Similar to this, the NLT of SLP-E shows improvement by 17%, 0.2%, 83%, and 13% in the SLP-R, SRR, PRLPRW, and PSSLP techniques, respectively. When we examine these data, it is evident that the suggested method performs better than the existing ones in both the safety period and network lifetime metrics. In addition, contrary to prior solutions where privacy strength is distance-dependent, the proposed technique demonstrates consistent privacy and network lifetime levels for any position of the source in the network. Therefore, the suggested approach achieves its main objectives of offering an improved safety period and NLT while maintaining uniform privacy levels.

- The proposed scheme’s (SLP-E) performance is compared to the existing schemes in terms of safety period, as illustrated in Figure 6. In configuring the simulation environment, we position the source nodes as follows: (200, 0), (550, 0), (900, 0), and (1050, 0). Notably, SLP-E outperforms existing SLP schemes in terms of safety period, and regardless of the source node’s location within the network, SLP-E consistently maintains a high level of privacy. This attribute of SLP-E is a result of its ability to adapt the routing protocol based on the source’s position within the network.
- It is seen that the proposed scheme performs equally well along with the SRR technique concerning network lifetime (NLT), when compared to other existing solutions. Nevertheless, our findings demonstrate that, on average, SLP-E achieves superior NLT performance in comparison to SRR. Notably, NLT in SLP-E remains consistently high across various network settings, as depicted in Figure 11. The suggested strategy remains the optimal choice for consistently achieving improved safety period and network lifetime (NLT).
- This study has additionally conducted a thorough analysis of the impact of sensor nodes’ radio range on various performance metrics of SLP. In order to gain a comprehensive understanding of how the proposed protocol (SLP-E) behaves under varying radio range settings, we conducted tests with the following radio ranges: 72, 77, 82, 87, 92, and 97. It becomes evident that radio range size has noticeable effects on the safety period, capture ratio, and network lifetime metrics.

Further, it has been noted that increased delays are required to obtain a safety period enhancement; this is considered as a limitation of this scheme. Despite the fact that these two metrics are trade-offs, future work can focus on improving SLP protection methods that take advantage of the optimization approach to reduce packet transmission latency. By using q-learning principles from the machine learning field, we intend to investigate multi-objective optimization of safety period, network life, energy consumption, and delay in the future.

## Figures and Tables

**Table 1:** Notations and descriptions.

Notations	Description	Notations	Description
$d_{0}$	Threshold distance	$E_{elec}=50$ n J/bit	Loss in transmission circuit
$e_{f_{s}}=$	Power amplification	l= 2048 bits	Packet’s length
10 PJ/bit/m${}^{2}$	in free space model		
$e_{amp}=0.00013$	Power amplification	*d*	Distance between transmitter
PJ/bit/m${}^{4}$	in fading model		and receiver
*R*	Radius of deployment area	$r_{s}$	Radio range of nodes
$P_{r}$	Probability of relaying	$H_{11}$	Hops in phase-1 scenario-1
$H_{12}$	Hops in phase-2 scenario-1	$H_{13}$	Hops in phase-3 scenario-1
$H_{14}$	Hops in phase-4 scenario-1	$H_{15}$	Hops in phase-5 scenario-1
$H_{t1}$	Max. no. of hops in scenario-1	θ	Hop length in angular walk
$H_{21}$	Hops in phase-1 scenario-2	$H_{22}$	Hops in phase-2 scenario-2
$H_{23}$	Hops in phase-3 scenario-2	$H_{24}$	Hops in phase-4 scenario-2
$H_{25}$	Hops in phase-5 scenario-2	$H_{t2}$	Max. no. of hops in scenario-2

**Table 2:** Summary of performance characterization.

Metrics vs. Protocol	Safety Period	Capture Ratio	Entropy	Energy Consumption	Delay	Network Lifetime
SLP-R	5024	−50	17,001	17,857	318	28
SRR	3185	−10	10,287	10,726	241	49.8
PRLPRW	2803	−48	10,551	7892	228	−18
PSSLP	5479	−72	20,701	22,432	393	33
SLP-E	6379	−60	22,996	29,923	463	50

## Data Availability

Data are contained within the article.
